# Risky Drinking Patterns Are Being Continued into Pregnancy: A Prospective Cohort Study

**DOI:** 10.1371/journal.pone.0086171

**Published:** 2014-01-15

**Authors:** Amy E. Anderson, Alexis J. Hure, Peta M. Forder, Jennifer Powers, Frances J. Kay-Lambkin, Deborah J. Loxton

**Affiliations:** 1 Priority Research Centre for Gender, Health and Ageing, School of Medicine and Public Health, University of Newcastle, Callaghan, New South Wales, Australia; 2 Priority Research Centre for Translational Neuroscience and Mental Health Research, University of Newcastle, Callaghan, New South Wales, Australia; 3 National Drug and Alcohol Research Centre, University of New South Wales, Randwick, New South Wales, Australia; Iran University of Medical Sciences, Iran (Islamic Republic of)

## Abstract

**Background:**

Risky patterns of alcohol use prior to pregnancy increase the risk of alcohol-exposed pregnancies and subsequent adverse outcomes. It is important to understand how consumption changes once women become pregnant.

**Objective:**

The aim of this study was to describe the characteristics of women that partake in risky drinking patterns before pregnancy and to examine how these patterns change once they become pregnant.

**Methods:**

A sample of 1577 women from the 1973–78 cohort of the Australian Longitudinal Study on Women’s Health were included if they first reported being pregnant in 2000, 2003, 2006, 2009 and reported risky drinking patterns prior to that pregnancy. Multinomial logistic regression was used to determine which risky drinking patterns were most likely to continue into pregnancy.

**Results:**

When reporting risky drinking patterns prior to pregnancy only 6% of women reported weekly drinking only, whereas 46% reported binge drinking only and 48% reported both. Women in both binge categories were more likely to have experienced financial stress, not been partnered, smoked, used drugs, been nulliparous, experienced a violent relationship, and were less educated. Most women (46%) continued these risky drinking patterns into pregnancy, with 40% reducing these behaviors, and 14% completely ceasing alcohol consumption. Once pregnant, women who binged only prior to pregnancy were more likely to continue (55%) rather than reduce drinking (29%). Of the combined drinking group 61% continued to binge and 47% continued weekly drinking. Compared with the combined drinking group, binge only drinkers prior to pregnancy were less likely to reduce rather than continue their drinking once pregnant (OR = 0.37, 95% CI  =  0.29, 0.47).

**Conclusions:**

Over a third of women continued risky drinking into pregnancy, especially binge drinking, suggesting a need to address alcohol consumption prior to pregnancy.

## Introduction

Heavy alcohol use during pregnancy can have detrimental effects, such as Fetal Alcohol Spectrum Disorders [1] and brain malformations. [2] However, the effects of low to moderate antenatal alcohol use are inconclusive, making it difficult to identify a safe level of use. [3]–[5] To complicate things further, it has been reported that the effects of alcohol vary based on the pattern of consumption, [6] such that binge drinking (i.e. four to five or more drinks per occasion) or drinking on a weekly basis (i.e. drinking at least one standard drink a day per week) should be investigated when assessing antenatal alcohol use.

Binge drinking episodes during pregnancy have been found to increase the risk of adverse outcomes such as poor neurodevelopment, [3] birth defects and growth restrictions, [7]–[9] mental health problems, [10] and fetal and infant mortality. [11]–[12] Other studies have not found a significant association between binge drinking and certain child outcomes, such as intelligence, attention and executive function. [13]–[14] Frequent (i.e. weekly) antenatal alcohol consumption may also lead to negative outcomes, as it has been found that as little as 70 grams of alcohol a week (one standard drink per day) can increase the risk of child behavioral problems. [6] Additionally, children’s IQ may be negatively affected by genetic variations linked to moderate antenatal alcohol intake of just one to six drinks per week during pregnancy. [15]

Considering the complexity regarding the effects of alcohol consumption during pregnancy and the inability to define a safe level of alcohol use, a number of alcohol guidelines worldwide have recommended abstinence for pregnant women. [16]–[19] One of the countries now recommending abstinence is Australia, [17] yet it is estimated that 72% of pregnant women consume alcohol. [20] Rates of alcohol use during pregnancy are also high in France [21] and the United Kingdom, [22]–[23] but not in other countries such as the United States [24] and Canada. [25] Previous research has found that alcohol use prior to pregnancy, particularly binge and weekly drinking, increase the risk of alcohol use during pregnancy. [20], [26]–[28] Binge and weekly drinking before pregnancy can therefore be considered risky drinking patterns, putting women at risk of experiencing an alcohol-exposed pregnancy and potential fetal harm.

It would be useful to establish if risky drinking patterns prior to pregnancy are modified once women become pregnant and if not, identify the characteristics of women engaging in these risky drinking patterns before pregnancy to enable early intervention. Some studies have reported the proportions of these drinking behaviors before and during pregnancy. [26], [28]–[29] However, those studies did not clarify if women made an effort to reduce their alcohol consumption by only ceasing these risky drinking patterns while still consuming some alcohol or if they completely stopped drinking. [27], [29]–[30] Given the move of many developed countries towards recommendations of abstinence during pregnancy, this is an important gap to fill. Further, these previous studies used retrospective measures of alcohol use prior to pregnancy, increasing the chances of recall bias. [27], [29]–[30] No Australian studies have yet investigated changes in risky drinking patterns from before pregnancy to pregnancy. As a high proportion of Australian women continue to use alcohol during pregnancy, there is a need to use prospective longitudinal data to investigate how risky drinking patterns change once Australian women become pregnant.

The aims of this study were to: define the characteristics of women partaking in risky drinking patterns prior to pregnancy; investigate if women modify their risky drinking patterns once they become pregnant; and identify risky drinking patterns prior to pregnancy that increase a woman’s risk of continuing the behavior into pregnancy.

## Methods

### Ethics Statement

Ethical clearance for the Australian Longitudinal Study on Women’s Health (ALSWH) was obtained from the Universities of Newcastle and Queensland, Australia (ethics approvals H0760795 and 2004000224). Women provided written informed consent to participate in the study.

### Sample

This study uses data from the ALSWH, which commenced in 1996. Using the national health insurance database which provides universal healthcare to all Australian citizens and permanent residents (Medicare), women were randomly sampled, with those from rural and remote areas sampled at double the rate of women from urban areas. Born between 1973–78, 1946–51, and 1921–26, three age cohorts of women were sent mailed invitations to participate. After the baseline survey in 1996, each cohort was mailed a survey on an approximately three-year interval basis. More detailed methods can be found on the longitudinal study’s website [31] or in previously published studies. [32]–[34].

**Figure 1:**
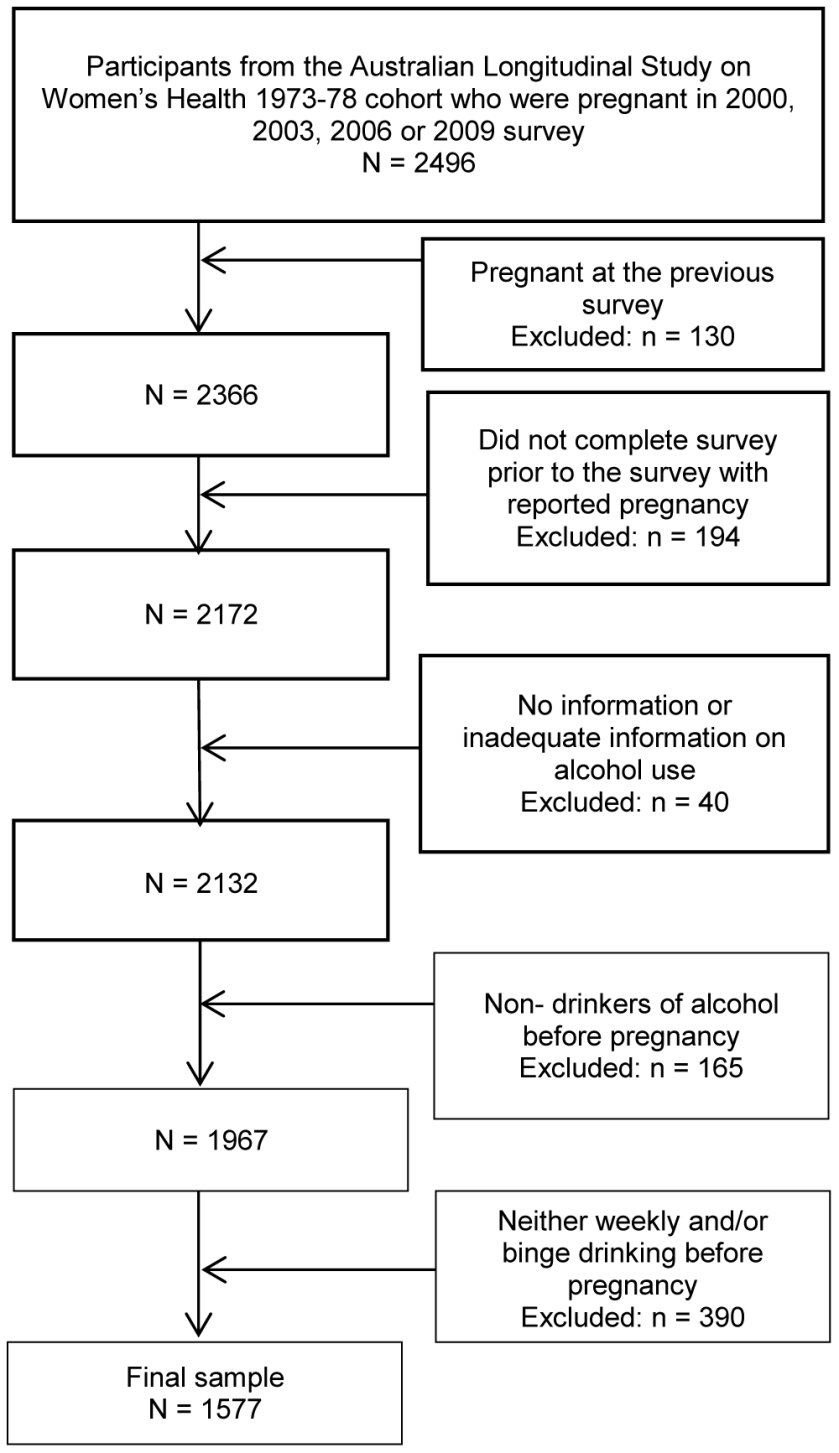
Flowchart of the sampling procedure. This includes the exclusion criteria used to draw the sample of women from the Australian Longitudinal Study on Women’s Health 1973–78 cohort.

The 1973–78 cohort data was used for this study. This cohort was broadly representative of similarly aged Australian women at the time of recruitment. [33] These women (aged 18–23 years in 1996) have completed five surveys to date – 1996, 2000, 2003, 2006, and 2009. Another survey was sent in 2012, but as data collection and quality checks occur over approximately 18 months, the dataset was still being finalized at the time of this study and could not be included in the analysis. Women who first reported a pregnancy at a survey time point after 1996 were eligible for inclusion into this study, with the survey prior to the index pregnancy being used to measure behaviors and characteristics of women before pregnancy. Only women that reported risky drinking patterns prior to pregnancy (i.e. weekly drinking, binge drinking, or both) were included in the analysis. Figure 1 presents the sampling strategy with exclusion criteria.

### Measures

Pregnancy status was determined using a prospective measure at every survey which asked “Are you currently pregnant?” Participant characteristics prior to pregnancy (i.e. the survey before the index pregnancy) were examined in relation to risky drinking patterns at that time. The sociodemographic and health-related characteristics that were measured at the survey before pregnancy included: participant’s age, partner status, highest educational attainment, area of residence, possession of private health insurance, level of stress about money to gauge income management, ever having experienced a violent relationship with a partner, having had a previous live birth, having had a Pap test in the last two years, ever having smoked or ever having used illicit drugs. The final response categories for these characteristics can be seen in Table 1.

**Table 1:** Characteristics of women according to their risky drinking patterns prior to pregnancy (N = 1577).

	Weekly only (n = 99)	Binge only (n = 725)	Weekly + Binge (n = 753)	Total	P
**Age (years, mean ± SD)**	28.64±2.74	25.60±3.50	27.07±3.37	26.49±3.51	0.56
	n (%)	n (%)	n (%)	n (%)	P
**Highest education attained**					
Higher school certificate (year 12) or less	19 (19.2)	298 (41.1)	199 (26.4)	516 (32.7)	<0.001
Trade/apprenticeship/certificate/diploma	16 (16.2)	199 (27.4)	164 (21.8)	379 (24.0)	
University or higher university degree	64 (64.6)	228 (31.4)	390 (51.8)	682 (43.2)	
**Area of residence**					
Major cities	64 (64.6)	328 (45.2)	418 (55.5)	810 (51.4)	<0.001
Inner regional	22 (22.2)	246 (33.9)	201 (26.7)	469 (29.7)	
Outer regional/remote/very remote	13 (13.1)	151 (20.8)	134 (17.8)	298 (18.9)	
**Private health insurance**					
No	44 (44.4)	460 (63.4)	381 (50.6)	895 (56.1)	<0.001
Yes	55 (55.6)	265 (36.6)	372 (49.4)	692 (43.9)	
**Income management stress**					
No stress or difficulty	85 (85.9)	562 (77.5)	602 (79.9)	1249 (79.2)	0.13
Stress and/or difficulty	14 (14.1)	163 (22.5)	151 (20.1)	328 (20.8)	
**Partner status**					
Not partnered	15 (15.2)	228 (31.4)	211 (28.0)	454 (28.8)	0.003
Partnered	84 (84.8)	497 (68.6)	542 (72.0)	1123 (71.2)	
**Violent relationship with a partner (ever)**					
No	95 (96.0)	622 (85.8)	663 (88.0)	1380 (87.5)	0.013
Yes	4 (4.0)	103 (14.2)	90 (12.0)	197 (12.5)	
**Pap test less than two years ago (n = 1573** * **)**					
No	21 (21.2)	162 (22.4)	157 (20.9)	340 (21.6)	0.79
Yes	78 (78.8)	562 (77.6)	593 (79.1)	1233 (78.4)	
**Illicit drug use – ever (n = 1575** * **)**					
No	62 (62.6)	315 (43.6)	204 (27.1)	581 (36.9)	<0.001
Yes	37 (37.4)	408 (56.4)	549 (72.9)	994 (63.1)	
**Smoking (ever)**					
No	74 (74.7)	391 (53.9)	385 (51.1)	850 (53.9)	<0.001
Yes	25 (25.3)	334 (46.1)	368 (48.9)	727 (46.1)	
**Previous live births**					
None	71 (71.7)	560 (77.2)	666 (88.4)	1297 (82.2)	<0.001
One or more	28 (28.3)	165 (22.8)	87 (11.6)	280 (17.8)	

Missing some cases.

Alcohol use items were measured at the survey when the woman was pregnant and at the survey prior to her pregnancy. Weekly drinking was measured by collapsing the answers to the question “How often do you usually drink alcohol?” into only two responses - ‘at least once a week’ versus ‘less than once a week’. The ‘less than once a week’ category was a combination of the response options ‘less than once a month’ and ‘less than once a week’. The ‘at least once a week’ category included response options ‘on 1 or 2 days a week’, ‘on 3 or 4 days a week’, ‘on 5 or 6 days a week’, and ‘every day’. Binge drinking was measured by the survey item “How often do you have five or more standard drinks of alcohol on one occasion?” with responses categorized into ‘never’ versus ‘ever’. The latter included the responses: ‘less than once a month’, ‘about once a month’, ‘about once a week’, and ‘more than once a week’. The usual quantity of alcohol consumption was measured by the item “On a day when you drink alcohol, how many standard drinks do you usually have?” Responses to this item were ‘1 or 2 drinks per day’, ‘3 or 4 drinks per day’, ‘5 to 8 drinks per day’, and ‘9 or more drinks per day’.

### Primary Outcome

The primary outcome was change in risky drinking patterns from before pregnancy to pregnancy. Risky drinking patterns before pregnancy were defined as drinking behaviors that had been found in previous studies to increase a woman’s risk of consuming alcohol during pregnancy. [20], [26]–[28] Risky drinking patterns were: weekly drinking only (i.e. drinking at least once a week, no binge drinking); binge drinking only (i.e. binge drinking, drinking less than once a week); or both weekly and binge drinking (i.e. drinking at least once a week and binge drinking).

The three levels used to categorize the primary outcome of change in risky drinking patterns from before pregnancy to pregnancy were ‘stopped’, ‘reduced’, or ‘continued’. A change to complete abstinence from alcohol during pregnancy was defined as ‘stopped’. A ‘reduced’ change varied per risky drinking group. For those in the binge only group, a change of drinking pattern from bingeing to alcohol use without bingeing was classified as ‘reduced’. A change from drinking at least once a week to drinking less than weekly was labeled as a ‘reduced’ change for the weekly drinking only group. For the combined drinking group (binge and weekly), the term ‘reduced’ referred to some alcohol use where either or both risky drinking patterns were ceased. Participants that continued their risky drinking patterns were used as the reference group in multivariate analyses. They were chosen as the reference group because they were considered to be most in need of intervention, as they did not report a change in risky alcohol consumption patterns once becoming pregnant.

### Statistical Analysis

All statistical analyses were run using SPSS (SPSS, version 19). Descriptive statistics were reported for socio-demographic and health-related characteristics in relation to the three risky drinking patterns prior to pregnancy (e.g. weekly only, binge only, or both binge and weekly) and were assessed using chi-square tests and Analysis of Variance (ANOVA), as appropriate. The distribution of usual quantity of alcohol use prior to pregnancy was calculated for each risky drinking pattern to examine drinking habits within groups. Characteristics that significantly differed between the three groups (p<0.05) were considered in the following multivariate analyses.

The association between risky drinking patterns prior to pregnancy and change in drinking behavior from before pregnancy to pregnancy was examined using multinomial logistic regression. The outcome for the regression was the change in drinking patterns, modeling the risk of stopping or reducing the risky drinking pattern versus continuing such behavior into pregnancy. Unadjusted odds ratios were initially calculated. Then the model was adjusted for participant characteristics, building the model by controlling for characteristics significantly related to risky drinking patterns prior to pregnancy. A final multinomial logistic regression model was conducted controlling for all significant characteristics. Although it was not a main focus of this analysis, the final model was adjusted to see if the change in Australian alcohol guidelines for pregnant women (i.e. 1992: no alcohol, 2001: low alcohol, 2009: no alcohol) [17], [35]–[36] impacted the relationship between risky drinking patterns prior to pregnancy and the change of drinking patterns once becoming pregnant.

## Results

Of the 1577 participants included in the analysis, 19% reported a pregnancy in 2000, 23% in 2003, 32% in 2006 and 26% in 2009. Ninety-nine (6%) reported that before pregnancy they consumed alcohol at least weekly without any binge drinking, 725 (46%) reported only binge drinking during this time, while 753 (48%) reported both weekly and binge drinking patterns prior to pregnancy. The majority (94%) of participants that were weekly drinking usually consumed no more than two drinks on a drinking day, with the remaining 6% reporting three to four drinks per drinking day. Of the participants in the binge only drinking group, on a drinking day 37% drank up to two drinks, 35% drank three to four drinks, while the remaining 28% drank five or more. The majority (51%) of participants in the combined drinking group reported drinking up to two drinks on a drinking day, with 36% drinking three to four and 13% drinking five or more drinks. Table 1 presents the participants’ characteristics prior to pregnancy in relation to these drinking patterns. Overall the women were mostly highly educated (43%), married or in a de facto relationship (71%), nulliparous (no previous live birth; 82%), and lived in major cities (51%) prior to pregnancy. Compared to women in the weekly drinking group, women in both binge groups (i.e. binge only and combined group) were more likely to have experienced a violent relationship, be nulliparous, have smoked and used illicit drugs, and were less likely to be highly educated, live in major cities, be partnered and have private health insurance.

Regardless of risky drinking patterns before pregnancy, fewer than 17% of the women completely stopped these behaviors once they became pregnant, with most women (46%) continuing these risky drinking patterns. Table 2 provides details of the changes in participants’ risky drinking patterns from before pregnancy to pregnancy. Most women (44%) who were only drinking weekly prior to pregnancy were likely to continue this behavior when pregnant, with 16% of this group completely abstaining from alcohol consumption while pregnant. The proportion of women who continued to binge drink only during pregnancy was higher (55%), with a similar proportion abstaining once pregnant (16%). Of the combined drinking group, 13% stopped consuming alcohol during pregnancy, with 41% reducing weekly drinking and 26% reducing binge drinking. Slightly less than half (47%) of the combined group continued weekly drinking, whereas 61% of this group continued binge drinking into pregnancy.

**Table 2:** Changes in risky drinking patterns from before pregnancy to pregnancy (N = 1577).

	Change in drinking patterns					
Drinking patterns before pregnancy	Stopped		Reduced		Continued	
	n	(%)	n	(%)	n	(%)
Weekly drinking only (n = 99)	16	(16.2)	39	(39.4)	44	(44.4)
Binge drinking only (n = 725)	114	(15.7)	212	(29.2)	399	(55.0)
Both weekly and binge drinking (n = 753)	95	(12.6)	377	(50.1)	281	(37.3)
Total	225	(14.3)	628	(39.8)	724	(45.9)

Table 3 contains the results for the multinomial logistic regression models assessing the association of risky drinking patterns prior to pregnancy and the change of such behaviors once women became pregnant. Compared to women that consumed alcohol through both weekly and binge drinking before pregnancy, those who binged only at that time were around 63% less likely to reduce rather than continue their risky drinking patterns when pregnant (AOR  =  0.37, 95% CI  =  0.29, 0.47). In other words, women who binged only were about two and a half times more likely to continue rather than reduce this behavior when compared to women in the combined drinking group. Women who were weekly drinking only rather than both binge and weekly drinking before pregnancy were found to be 42% less likely to reduce rather than continue (i.e. 1.7 times more likely to continue rather than reduce) their drinking behavior once illicit drug use and smoking status were taken into account (AOR  =  0.58, 95% CI  =  0.36, 0.94). There was no evidence of a difference between drinking pattern groups before pregnancy on the likelihood of stopping all alcohol consumption in pregnancy. The alcohol guidelines that were in place during the reported pregnancies did not significantly alter the relationship between risky drinking patterns before pregnancy and the change of these patterns once becoming pregnant [results not shown].

**Table 3:** The association of risky drinking patterns prior to pregnancy with changes in these patterns during pregnancy.

	Unadjusted		Model 1^a^		Model 2^b^		Model 3^c^		Model 4^d^		Final model^e^	
	OR	(95% CI)	OR	(95% CI)	OR	(95% CI)	OR	(95% CI)	OR	(95% CI)	OR	(95% CI)
**Reduced (versus continued)**												
Weekly + Binge	1		1		1		1		1		1	
Weekly only	0.66	(0.42,1.04)	0.66	(0.42,1.04)	0.67	(0.42,1.06)	0.54	(0.34,0.87)	0.70	(0.44,1.12)	0.58	(0.36,0.94)
Binge only	0.40	(0.32,0.50)	0.40	(0.31,0.50)	0.39	(0.31,0.50)	0.36	(0.29,0.46)	0.41	0.33,0.52)	0.37	(0.29,0.47)
**Stopped (versus continued)**												
Weekly + Binge	1		1		1		1		1		1	
Weekly only	1.08	(0.58,2.00)	1.12	(0.60,2.07)	1.11	(0.60,2.07)	0.99	(0.53,1.85)	1.16	(0.62,2.15)	1.13	(0.60,2.14)
Binge only	0.85	(0.62,1.16)	0.80	(0.58,1.11)	0.84	(0.61,1.14)	0.82	(0.60,1.13)	0.88	(0.65,1.21)	0.81	(0.60,1.16)

^a^ Adjusted for highest education attained, area of residence, private health insurance.

^b^ Adjusted for partner status, violent relationship with a partner (ever).

^c^ Adjusted for illicit drug use (ever), smoking (ever).

^d^ Adjusted for previous live births.

^e^ Adjusted for highest education attained, area of residence, private health insurance, partner status, violent relationship with a partner (ever), illicit drug use (ever), smoking (ever), and previous live births.

## Discussion

By utilizing data from a population-based prospective cohort study, the results provide a strong level of evidence to suggest that Australian women who participate in risky drinking patterns before pregnancy are likely to continue these drinking patterns into pregnancy. There is only a small likelihood that these women will completely abstain from alcohol during pregnancy. Less than one in five women stopped consuming alcohol once becoming pregnant, with no difference in stopping between the three drinking categories. However, a substantial proportion of women made the move in the right direction by reducing these risky drinking patterns when pregnant. Interestingly, women partaking in both binge and weekly drinking were more likely to reduce their drinking compared to those who only did one or the other. This may be due to the fact that they had more opportunity to reduce as there were two behaviors they could change rather than just one. However, further investigation is needed to understand why this was the case.

Although some women took a positive step in reducing risky alcohol patterns once they were pregnant, women who participated in binge drinking prior to pregnancy were the least likely to do so. Even the women who partook in both risky drinking patterns (i.e. weekly and binge) prior to pregnancy were less likely to reduce their binge drinking rather than their weekly drinking. These findings lend support to previous research from France which found that binge drinking was more common than weekly drinking in pregnant women, [21] perhaps due to limited change from binge drinking patterns prior to pregnancy. The current findings may be reflective of the reported permissive view of binge drinking among young women, particularly in the Australian context, which conceptualizes binge drinking as an enjoyable behavior that plays a meaningful role in socialization. [37] The documented ill effects of binge drinking are consistently being demonstrated [38] and this study adds to this list the increased risk of an alcohol-exposed pregnancy.

Women in the current study who binge drank prior to pregnancy appeared to be of a lower socio-economic status as reflected by their lower education status and lack of private health insurance. Binge drinking in this group could be due to a difference in knowledge and views, as previous examination of women’s perceptions of safe levels of alcohol consumption found that the mean number of alcoholic drinks believed to be acceptable on any one occasion seemed to reduce with higher socioeconomic advantage. [39] Additionally, it has been reported that Australian women with lower education levels are less knowledgeable about the negative impacts of alcohol use during pregnancy. [40] These women may therefore require a more targeted intervention aimed at increasing education and motivating change in alcohol use to achieve abstinence or at the very least a reduction of binge drinking in response to pregnancy. Previous research has found that motivational interviewing that focused on contraception and alcohol use was effective in reducing the risk of alcohol-exposed pregnancies among women of childbearing age. [41]–[42] Considering that over 50% of Australian women have reported experiencing an unplanned pregnancy, [43] it is critical that prevention strategies be employed as early as possible either through clinical intervention or public health schemes.

Also of interest was the finding that women who consumed alcohol before pregnancy through weekly drinking only were found to be significantly less likely to reduce their drinking behavior only after illicit drug use and smoking status were taken into account. The findings from this group need to be interpreted with caution given the small sample size (n = 99). Previous research found that the chances of continuing concurrent alcohol use and smoking into pregnancy increased if women were heavier smokers prior to pregnancy. [44] This may be due to the fact that women who smoke have been found to have more tolerant attitudes towards drinking during pregnancy. [40] Therefore, drinking behavior should not be assessed in isolation, but rather routinely within the context of other behaviors when trying to identify women at risk of continuing their risky drinking behavior into pregnancy. These findings also lend weight to healthcare professionals’ previous suggestions that alcohol use be assessed along with other health behaviors. [45]

### Limitations

The use of a self-report questionnaire lends itself to the potential for social desirability bias. However, a previous study found that pregnant women accurately reported their smoking, a behavior considered socially unacceptable, when compared to biological measurements. [46] Additionally, self-reported alcohol use by pregnant women has been found to be better than medical records for assessing antenatal alcohol consumption. [47] Another limitation is that a validated instrument was not utilized to assess alcohol use. The alcohol questions did assess frequency, quantity and binge drinking, which are similar to the Alcohol Use Disorders Identification Test consumption items (AUDIT-C), [48] which has been found to be effective in screening alcohol use among pregnant women. [49] The main difference was that this cohort study assessed alcohol in terms of the ‘usual’ amount that was consumed rather than in the previous year as assessed by the AUDIT-C, which may have been beneficial in reducing recall bias. The ALSWH utilized prospective measures of alcohol use and pregnancy, rather than retrospectively collecting data in between surveys. This limits recall bias, but also means that drinking behavior in between survey time points could not be assessed. Therefore, pregnancies were limited to those that occurred at the specified survey time points, where alcohol use during pregnancy could be measured. Alcohol use at the previous survey was considered as one indicator of the women’s alcohol use prior to pregnancy regardless of whether this changed over time. Participants were not asked whether they had planned their pregnancies. However, previous studies have found that whether a pregnancy is planned or unplanned does not impact drinking behavior in the recognized phase of pregnancy [29]–[30], which is the phase examined by this study.

### Practice Implications

The findings of this study highlight the need for a primary prevention strategy to reduce prenatal alcohol use by addressing risky drinking patterns, particularly binge drinking, prior to conception. This study provides further support to existing clinical guidelines which promote alcohol consumption being addressed before pregnancy occurs. [50] There is a dearth of evidence when it comes to assessing interventions to reduce the risk of antenatal alcohol use before pregnancy. [51] However, using motivational interviewing to reduce risky alcohol consumption and increase contraception among women of childbearing age has been found effective in reducing the risk of alcohol-exposed pregnancies. [41]–[42] More research is needed to identify which strategies would be most effective in reducing women’s risky drinking patterns prior to pregnancy.

## Conclusion

The majority of women with risky drinking patterns before pregnancy continued these behaviors once they became pregnant. Although a number of women modified their drinking habits by reducing risky drinking patterns, less than one in five women in this sample completely abstained from alcohol once becoming pregnant, as currently recommended by a number of guidelines worldwide. [16]–[19] The substantial number of women that continued these behaviors into pregnancy, particularly those who binge drank, suggests that more needs to be done to address risky drinking behaviors in women of childbearing age in an effort to avoid alcohol use during pregnancy.
